# Social-ecological factors influencing loneliness and social isolation in older people: a scoping review

**DOI:** 10.1186/s12877-023-04418-8

**Published:** 2023-11-09

**Authors:** Drew Eleanor Meehan, Anne Grunseit, Jenna Condie, Neta HaGani, Dafna Merom

**Affiliations:** 1 School of Health Sciences, Western Sydney University, Campbelltown, Australia; 2 School of Public Health, Faculty of Health, University of Technology Sydney, Sydney, Australia; 3 School of Social Sciences, Western Sydney University, Paramatta, Australia; 4 Prevention Research Collaboration, School of Public Health, University of Sydney, Camperdown, Australia

**Keywords:** Loneliness, Social isolation, Social-ecological model, Ageing, Community, Societal, Scoping review

## Abstract

There are growing calls from researchers and policy makers to redefine loneliness and social isolation (SI) as public health issues, and to move towards a transdisciplinary, systems-based approach, due to their association with significant health risks, particularly in older people. Research about loneliness and SI in older people has typically adopted a narrow focus, evaluating effects of individual and inter-personal factors on these experiences. Less is known about the community and societal influences that may be used to inform public health interventions. We conducted a scoping review applying Joanna Briggs Institute methodology and the social-ecological model framework in order to: i) identify the available evidence for the influence of the community and societal factors on loneliness and SI as experienced by older people; ii) examine how quantitative research about community- and societal-level factors of loneliness and SI in the older population is conducted; and iii) identify current knowledge gaps in relation to the use of the social-ecological model in this area. A total of 52 articles from 30 countries met the inclusion criteria, including 33 observational studies, primarily cross-sectional (88%), and 19 interventions, mostly (89%) pre-post evaluations. The majority of included articles measured loneliness only (*n* = 34, 65%), while 11 measured both loneliness and SI (21%). To measure these outcomes validated scales were frequently used. Eighteen community/societal factors were investigated in relation to loneliness and/or SI, most commonly neighbourhood safety, access to public third-places and cultural practices. Three societal-level interventions were found: two campaigns to reduce ageism and one which explored the impact of free public transport. Community-based interventions were either educational or enlisted volunteers to foster connections. There is a need for longitudinal studies to better understand the mechanisms through which community- and societal- level factors affect loneliness and SI, which in turn will guide interventions that utilise the social-ecological framework for these issues.

## Background

Loneliness and social isolation (SI) are well-established as factors contributing to the development of a range of chronic health conditions including dementia, cardiovascular diseases and depression [1, 2]. Articles have found that the health implications of loneliness and SI are comparable to well-accepted risk factors like obesity and tobacco usage [3]. The increase in chronic illnesses as a result of loneliness and SI, combined with the more frequent use of health care services by those who are lonely and/or isolated [4], has both health and economic implications for society [5].

Loneliness, also known as subjective isolation, is defined as “the subjective unpleasant or distressing feeling of a lack of connection to other people, along with a desire for more, or more satisfying, social relationships” [6]. Conversely, SI, or objective isolation, refers to “having objectively few social relationships, social roles and group memberships, and infrequent social interaction” [6]. Although research on loneliness and SI has previously been carried out in various ways, NEG Newall and VH Menec [8] argue the two concepts should be studied together and understood as entwined. It is also difficult to separate or draw a boundary between where objective ends and subjective begins.

Several systematic reviews have investigated the risk factors of loneliness and SI in the older population, with increasing age one of the most cited risk factors [9]. While loneliness and SI are not exclusive to older people, a large proportion of those at risk of or experiencing loneliness and SI are from the older population [10], particularly those over the age of 70 years old [11]. Other commonly cited risk factors include gender, with women at greater risk of loneliness and men more at risk of SI [9] and older people with a lower level of educational attainment are at greater risk of both [12]. Older people with functional and cognitive impairments are also likely to experience an increased risk of both loneliness and SI [13].

It should be noted that the above mentioned well-explored risk factors are all primarily focused on the individual and not their broader social context. One of the reasons for the individual-focus approach is that loneliness and SI research has typically not been viewed as a public health issue [14]. However, growing evidence of the health implications of loneliness and SI, coupled with the increasing prevalence in Western societies, makes it clear that a public health approach that includes preventative measures must be included in the research discourse [4, 15].

There are growing calls from researchers and policy makers to redefine loneliness and SI as a public health issue, and to move towards a more transdisciplinary, systems-based approach [16]. Approaching the issues of loneliness and SI through the social-ecological lens allows for a more comprehensive and systematic analysis of the factors that influence loneliness and SI in older people. In doing so, more effective ways of alleviating loneliness and SI in this population may become available [17]. A person-centred approach to reducing loneliness and SI, such as social prescribing, is the gold standard of loneliness and SI interventions, but can be resource-intensive [18]. It is possible that altering, through systematic interventions, the community and societal factors, once identified, would be more effective in controlling loneliness and SI at the population level than the previous individualised interventions.

### Conceptual framework

We propose that an appropriate framework to apply to the issues of loneliness and SI in a public health context may be the social-ecological model [19]. This model represents a need to address the complexities of individuals and the world around them, and the use of the social-ecological model signals a departure in public health research from the increasingly outdated biomedical approach, to a more holistic method of addressing public health problems [14]. The social-ecological model has been used effectively to provide solutions to other pressing health issues such as maternal and child health, tobacco control, and physical inactivity [14].

There are many iterations of the social-ecological model, but the one used for this project is the World Health Organization endorsed model, initially proposed by L Dahlberg and EG Krug [19]. This version proposes four nested levels of interaction: the individual, the interpersonal, the community and the societal. Individual and interpersonal factors of loneliness and SI which make up the ‘micro level’, are well researched and have been reviewed systematically previously [9, 12, 13, 20]. Examples of these factors include socio-demographic characteristics, health status and health-related behaviours or their antecedents (e.g., knowledge, attitudes) [20]. The community and societal level interactions are less well-researched, and as such, are the focus of this review.

The concept of what constitutes community can differ depending on context. Community may differ within the bounds of whether it is physical or virtual, the level of geography, or the units of analysis [21]. For this review all modes of community were included, if they were defined as such in the source article, with the most common type of community researched being the neighbourhood which is the geographical area in which a person resides [22]. Community factors may impact a person’s health through the local environment such as the types of organisations that exist in the community, public spaces, and the cohesiveness of the neighbourhood [23]. This level may include local businesses, neighbourhood parks, and volunteering opportunities [24]. Societal-level factors, on the other hand, may influence loneliness and SI through social and economic policy or regulations, culture, and other social norms. Examples of societal influences include the media coverage of an issue, health-promoting legislation, shared ideas and religious beliefs [25].

To address loneliness and SI at all levels there is a need to evaluate what literature exists on the community and societal context that may affect loneliness and SI in older people. Therefore, this review has three main aims which are: To systematically identify available evidence for what the influential community and societal factors on loneliness and SI are as experienced by older people, and what their effects are; to examine how research about community and societal factors is conducted; and to identify knowledge gaps in relation to loneliness and SI through the lens of the social-ecological model.

## Method

The protocol for this scoping review was registered prospectively before commencing the searches on Open Science Framework: https://osf.io/wbp23/?view_only=b515662e37b44abe86bbba139d5e462f.

### Study design

To meet the aims of this review, a scoping review methodology was selected as this is quite a broad topic, and because there was a need to map and clarify the key components of the social-ecological model in relation to loneliness and SI [26]. We followed common methodology as determined by AC Tricco, E Lillie, W Zarin, KK O'Brien, H Colquhoun, D Levac, D Moher, MDJ Peters, T Horsley, L Weeks, et al. [27] for this scoping review. Criteria for reported items as determined by the Preferred Reporting Items for Systematic reviews and Meta-Analyses- extension for Scoping Reviews (PRISMA-ScR) were met [28].

### Search strategy

We devised our search strategy in line with the population, concept and context (PCC) framework from the Joanna Briggs Institute, in collaboration with a health science research librarian [28]. We searched five databases; CINAHL Plus, Embase, MEDLINE (OVID), PsycInfo, and Web of Science, between the 1^st^ and 30^th^ of August 2022, and the search strategy was adapted to meet the truncation and Boolean operations of each database as appropriate. The search strategy for Medline is available in Table 1.

**Table 1:** Search strategy used for OVID Medline database

Line #	Search terms
1	(old* OR senior* OR elder* OR geriatric*).ti,ab
2	aged/
3	1 OR 2
4	(lonel* OR social-isolation OR social-support OR social-deprivation).ti,ab
5	social-isolation/ OR loneliness/
6	4 OR 5
7	(communi* OR neighbo?r* OR cultur* OR polic* OR built environment* OR soci?-ecolog* OR environment* OR societ* OR ecologic?-model).ti,ab
8	*social-environment/ OR health-policy/ OR *residence-characteristics/
9	7 OR 8
10	3 AND 6 AND 9

Wildcards (*, ?) used for truncation and alternate spellings. Medical Subject Headings (MeSH) terms denoted by ‘/’. Boolean operators used as denoted in search term lines

Search results were uploaded into Endnote [29] and duplicates were removed. Covidence [30] was used for title and abstract screening by one reviewer. Full texts were screened for relevance by two reviewers and any conflicts were resolved by a third reviewer.

### Eligibility criteria

Inclusion and exclusion criteria were determined using the PCC framework. Included articles must be researching an older population, consisting of people aged 50 and older, with a mean age over 60 living in the community, not including older adults living in an institution. Articles must include a measure for subjective and/or objective isolation, and they must investigate community or societal level variables or interventions incorporating community or societal approaches. We excluded articles if only individual and interpersonal variables were addressed. We included published quantitative or mixed methods articles which used an interventional or observational methodology in this scoping review, excluding commentaries and reviews. All included articles were published in English, and no date restrictions were applied.

### Data charting

Critical analysis was conducted using the appropriate tools from the JBI suite of critical appraisal tools [31]. The critical appraisal and data extraction were completed concurrently using an Excel spreadsheet [32]. Detailed data extraction criteria were developed to maintain consistency when data charting and were tested on a subsample of included articles to determine the applicability of the criteria. Charted data included name of first author, primary affiliation of first author, publication year, conflicts of interest, funding source, aim of study, study design as reported by the authors, method of data collection, recruitment method, population characteristics including the included age range, mean age, female percentage, country where conducted, and the specific location if mentioned. The main outcome of interest, and the measurement tool used were recorded, as well as any co-variates mentioned by the authors. For intervention articles a description of the intervention was summarised, whether there was a control group and whether their treatment differed. For observational articles the exposure variable was recorded according to whether it was a community or societal level variable. The extraction also included any relevant findings and recommendations made by the authors for future research. A record of comments made by the extractor was also kept.

## Results

### Article characteristics

**Fig. 1:**
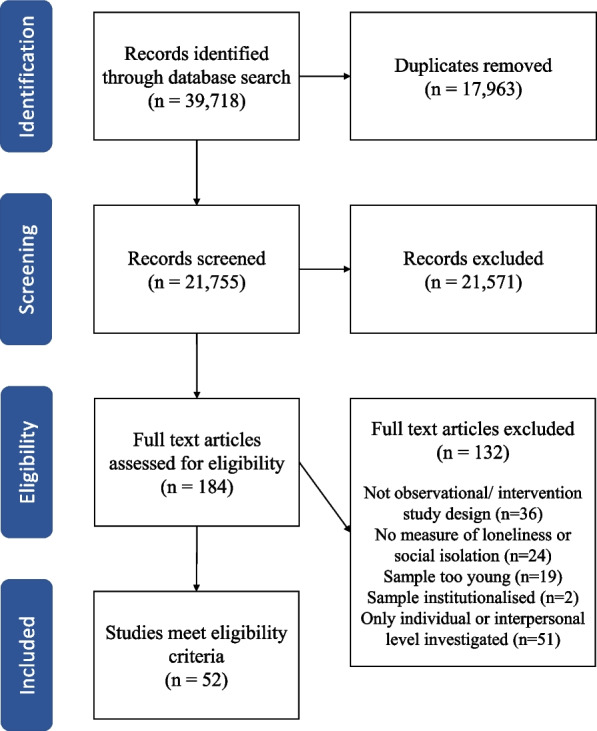
Flowchart of the screening process as per PRISMA recommendations [33]

As per the PRISMA diagram (Fig. 1) 39,718 records were returned from the database searches [33]. After duplicate removal there were 21,755 results to screen for title and abstract. From this, 184 articles were identified for full-text screening and reasons for exclusions were recorded. There were 52 articles identified as meeting all eligibility criteria and were therefore included in the scoping review [34–85].

Of the 52 included studies, 63 per cent (*n* = 33) were observational [34, 35, 39–43, 45, 48–55, 57–59, 62–66, 68, 71, 74, 78, 80, 81, 83–85], and 37 per cent (*n* = 19) were interventional [36–38, 44, 46, 47, 56, 60, 61, 67, 69, 70, 72, 73, 75–77, 79, 82]. Of the articles detailing observational studies, the majority (88%, *n* = 29) used a cross-sectional design [34, 35, 39–41, 43, 45, 48–52, 54, 55, 57–59, 62–66, 68, 71, 74, 80, 81, 83, 85], and only four articles utilised a longitudinal design [42, 53, 78, 84]. Of the interventional articles two were randomised controlled trials [61, 76], and 17 utilised a pre-post evaluation design with no comparison group [36–38, 44, 46, 47, 56, 60, 67, 69, 70, 72, 73, 75, 77, 79, 82]. Further descriptions of the included articles are shown in Table 2.

**Table 2:** Summary of charted data

Source	Topic focus	Sample	Subjective Isolation Measure and Tool	Objective Isolation Measure and Tool	Data Collection Method	Community Factors Being Investigated	Societal Factors Being Investigated	Co-variates adjusted for	Recommendations
**Observational**									
**Cross Sectional**									
(Ajrouch, 2008) [34]	Acculturation to explain differences in social isolation and loneliness	Country: United States of America Mean age: 69 % Female: 55.5 Sample size: 101	Loneliness, Single Question 'In the last two weeks, how often—if at all—did you feel lonely?'	Social Isolation, Hierarchical mapping technique- participants to rate level of relationships	Face-to-Face Interview Surveys	NS	Migration, Cultural Practices	Age, Education, Ethnicity, Gender, Marital Status, Physical Limitations	Investigate how immigrant status influences social isolation and loneliness over the life course
(Bai et al. 2021) [35]	Social capital and loneliness and the effect of social capital, demographic factors and health-related factors on loneliness	Country: China Mean age: 71.2 Sample size: 1810	Loneliness, Single Question 'Do you have the feeling of loneliness?'	NS	Face-to-Face Interview Surveys	Social Cohesion	NS	Age, Chronic Condition, Education, Gender, Marital Status, Living Arrangement, Physical Limitations	Longitudinal data or a randomised control trial
(Beech & Murray, 2013) [39]	Social engagement and its link with community attachment	Country: UK Mean age: 71.6 % Female: 76.9 Sample size: 65	Loneliness, DJG-LS-11	NS	Self-Complete Survey	NS	Neighbourhood Belonging	Physical Limitations, Religion	Need novel approaches to investigating loneliness
(Beer et al. 2016) [40]	Regional variations and incidence of social isolation	Country: Australia Sample size: 1682	Loneliness, Single Question 'During the past four weeks I felt isolated from other people.'	Social Isolation, Friendship Scale	Self-Complete Survey	Rurality	NS	Physical Limitations, Religion	No future research recommendations
(Beere et al. 2019) [41]	Socio-spatial trends for loneliness	Country: New Zealand/ Aotearoa Sample size: 52,973	Loneliness, Single Question	NS	Face-to-Face Interview Surveys	Rurality	NS	Physical Limitations, Religion	Need data at a more granular level
(Burholt et al. 2018) [43]	Social networks that are most vulnerable to loneliness	Country: UK % Female: 50 Sample size: 815	Loneliness, DJG-LS-6	NS	Face-to-Face Interview Surveys	NS	Cultural Practices	Age, Ethnicity, Gender, Marital Status, Self-Rated Health	More evidence about indigenous and migrant populations from collectivist cultures
(Cao et al. 2020) [45]	Access to outdoor space and buildings and social or community events, and perceived disconnection	Country: United States of America Mean age: 65.01 % Female: 63.5 Sample size: 346	Subjective Isolation, Single Question 'I frequently feel disconnected from my community.'	NS	Self-Complete Survey	Public Third-Places	NS	Age, Gender, Home accessibility, Income, Living Arrangement, Self-Rated Health	More research about older peoples existing community knowledge before designing interventions
(Dahlberg et al. 2022) [48]	Associations between indicators of social exclusion and loneliness	Country: Denmark, Finland, Norway, and Sweden Sample size: 7755	Loneliness, Single Question ‘how much of the time during the past week have you felt lonely’	NS	Face-to-Face Interview Surveys	Neighbourhood Safety	Political Participation	Age, Education, Gender, Self-Rated Health, Physical Limitations	Longitudinal data and more prospective designs to provide evidence for causal links
(De Jong Gierveld et al. 2015) [49]	Loneliness of immigrants compared to native-born people in an ecological model	Country: Canada Sample size: 3692	Loneliness, DJG-LS-6	NS	Face-to-Face Interview Surveys	Social Cohesion	Cultural Practices	Gender, Marital Status, Self-Rated Health	No future research recommendations
(De Koning et al., 2017) [50]	Predictors of loneliness and social isolation	Country: UK Mean age: 71.5 % Female: 57.9 Sample size: 884	Loneliness, Single Question 'I experience a general sense of loneliness'	Social Isolation,3 Item Scale	Face-to-Face Interview Surveys	Transport Access	NS	Age, Gender	No future research recommendations
(Diaz et al. 2019) [51]	Factors beyond immigration of loneliness among ethnic minority elders	Country: Canada % Female: 67.3 Sample size: 123	Loneliness, UCLA-LS-21	Social Network, LSNS-R-12	Self-Complete Survey	NS	Cultural Practices	Education, Employment, English Proficiency, Income	Investigate the relationship between unfulfilled expectations of social network and feelings of loneliness among ethnic older people
(Domenech-Abella et al. 2020) [52]	The role of built environment in loneliness and depression	Country: Finland, Poland, Spain % Female: 54.9 Sample size: 5912	Loneliness, UCLA-LS-3	NS	Face-to-Face Interview Surveys	Walkability, Public Third-Places	NS	Chronic Condition, Education, Gender, Marital Status	Longitudinal data to provide evidence for causality
(Gibney et al. 2019) [54]	Age-friendliness of local environments and self-reported loneliness	Country: Ireland % Female: 52.7 Sample size: 10,540	Loneliness, UCLA-LS-5	NS	Face-to-Face Interview Surveys	Accessible Services, Walkability, Transport Access	Perceptions Of Ageism	Age, Education, Gender, Income, Marital Status, Living Arrangement, Self-Rated Health	Longitudinal data for the relationship between loneliness and environment
(Glass, 2020) [55]	The intersection of loneliness and sense of community	Country: United States of America Mean age: 72 % Female: 72 Sample size: 86	Loneliness, UCLA-LS-3	NS	Self-Complete Survey	Neighbourhood Satisfaction	Neighbourhood Belonging	Physical Limitations, Religion	Need more research on the intersection of cohousing, age, gender, and loneliness
(Haslam et al. 2022) [57]	Social group memberships and the wellbeing of older immigrants	Country: Australia Mean age: 80.33 % Female: 82.35 Sample size: 102	Loneliness, UCLA-LS-3	NS	Self-Complete Survey	NS	Cultural Practices	Age, English Proficiency, Religion	Evidence for the efficacy of social groups in reducing immigrant loneliness across different groups and ages
(Henning‐Smith et al. 2019) [58]	Rurality and social isolation	Country: United States of America Mean age: 71 % Female: 50 Sample size: 2439	Loneliness, Loneliness Scale (3 item not validated)	NS	Face-to-Face Interview Surveys	Rurality	Migration	Age, Education, Ethnicity, Religion	Research how to facilitate connections between older adults in urban areas
(Hodgkin et al. 2018) [59]	Ecological model of wellness	Country: Australia Mean age: 75.92 % Female: 64.9 Sample size: 266	Loneliness, DJG-LS-6	Social Network, Single Question 'number of friends and family members'	Telephone Survey Interview	Accessible Services, Neighbourhood Satisfaction, Neighbourhood Safety	NS	Age, Gender, Income, Marital Status, Physical Limitations, Mental Health	Need for more research measuring wellness in older people
(Klok et al. 2017) [62]	Transnational sense of belonging as a dimension of belonging	Country: Netherlands Mean age: 60.9 Sample size: 461	Loneliness, DJG-LS-11	NS	Face-to-Face Interview Surveys	NS	Migration, Cultural Practices	Age, Education, Employment, Ethnicity, Gender, Marital Status, Self-Rated Health, Physical Limitations	Need for more research understanding how transnational belonging relates to wellness in older migrants
(Lam & Wang, 2022) [64]	Characteristics of the built environment and loneliness	Country: Australia Mean age: 62.9 % Female: 53 Sample size: 298	Loneliness, Single Question 'I often feel very lonely.'	NS	Face-to-Face Interview Surveys	Neighbourhood Disadvantage, Open Green Spaces, Neighbourhood Density	Housing Diversity	Education, Ethnicity, Gender, Income, Marital Status, Self-Rated Health, Children	Need for more research using the built environment as a level for increasing older people's connections
(Lam, 2022) [63]	Ethnic–migrant backgrounds and loneliness dependent on neighbourhood	Country: Australia Sample size: 22,183	Loneliness, Single Question 'I often feel very lonely.'	NS	Self-Complete Survey	Neighbourhood Density, Neighbourhood Satisfaction, Neighbourhood Safety	Migration	Age, Education, Gender, Income, Marital Status, Self-Rated Health	Research directly capturing social experiences and neighbourhood safety satisfaction for migrants from non-English speaking countries
(Lane et al. 2020) [65]	Neighbourhood destinations for socializing and social health	Country: Singapore Mean age: 67.98 % Female: 53.21 Sample size: 981	NS	Social Health, LSNS-6	Face-to-Face Interview Surveys	Neighbourhood Density, Walkability, Public Third-Places	NS	Age, Chronic Condition, Education, Ethnicity, Gender, Marital Status, Living Arrangement	Research types of activities that happen in third places as possible explanations for connections
(Lee, 2022) [66]	Volunteer work and loneliness	Country: United States of America Mean age: 75.94 % Female: 59.4 Sample size: 9944	Loneliness, UCLA-LS-3	NS	Self-Complete Survey	Public Third-Places	NS	Age, Education, Employment, Ethnicity, Gender, Marital Status, Living Arrangement, Self-Rated Health, Physical Limitations, Religion	Need more research to understand how socio-demographic and culture influence the association between volunteer work and loneliness
(Moorer & Suurmeijer, 2001) [68]	A neighbourhood effect for social network size and loneliness	Country: Netherlands Mean age: 74.6 Sample size: 723	Loneliness, DJG-LS-11	Social Network, ‘social network delineation questionnaire’	Self-Complete Survey	Public Third-Places, Neighbourhood Safety	NS	Physical Limitations, Religion	No future research recommendations
(Park et al. 2021) [71]	Age friendly environments and loneliness	Country: South Korea Mean age: 69.78 % Female: 48.2 Sample size: 353	Loneliness, UCLA-LS-3	NS	Face-to-Face Interview Surveys	Open Green Spaces, Walkability, Transport Access, Public Third-Places, Neighbourhood Safety	Perceptions Of Ageism	Chronic Condition, Education, Gender, Income, Marital Status, Self-Rated Health,	Need longitudinal data on the effect of Age Friendly Environments on loneliness and depressive symptoms
(Rezaeipandari et al. 2020) [74]	Social participation and sense of loneliness	Country: Iran Mean age: 70 % Female: 68 Sample size: 200	Loneliness, Social and Emotional Loneliness Scale	NS	Face-to-Face Interview Surveys	Transport Access, Public Third-Places, Neighbourhood Safety	NS	Education, Employment, Gender, Income, Marital Status, Living Arrangement	Need for more data allowing for causal inferences
(Stephens & Phillips, 2022) [80]	Perceived neighbourhood environment and emotional and social loneliness and the mediating effects of social networks	Country: New Zealand/ Aotearoa Mean age: 75 % Female: 53.2 Sample size: 917	Loneliness, DJG-LS-6	NS	Self-Complete Survey	Accessible Services, Social Cohesion, Neighbourhood Safety	Neighbourhood Belonging	Gender, Marital Status, Physical Limitations	Need more social structures to encourage natural social opportunities
(Timmermans et al. 2021) [81]	Objectively measured social and physical neighbourhood characteristics and loneliness	Country: Netherlands Mean age: 72.8 % Female: 49 Sample size: 1959	Loneliness, DJG-LS-6	NS	Self-Complete Survey	Public Third-Places, Neighbourhood Safety	Social Security Recipients	Age, Education, Gender, Income, Marital Status	More data examining environmental characteristics on loneliness in older adults
(Woolham et al. 2013) [83]	Factors associated with loneliness	Country: UK Sample size: 1558	Loneliness, Single Question 'Do you ever feel lonely and wish you had more company?'	NS	Self-Complete Survey	Transport Access, Neighbourhood Safety	NS	Age, Employment, Ethnicity, Income, Self-Rated Health	Qualitative research on groups of people underrepresented in community surveys
(Zhang & Lu, 2022) [85]	Financial status as a moderator of neighbourhood environment and loneliness	Country: China Mean age: 68.4 % Female: 55.8 Sample size: 459	Loneliness, DJG-LS-6-Chinese	NS	Face-to-Face Interview Surveys	Neighbourhood Safety	Neighbourhood Belonging	Age, Chronic Condition, Education, Gender, Marital Status, Living Arrangement, Physical Limitations	Examine the relationship between loneliness and the neighbourhood environment in rural areas
**Cohort**									
(Beller & Wagner, 2020) [42]	Individualism /collectivism and loneliness	Country: Austria, Belgium, Switzerland, Czech Republic, Germany, Denmark, Estonia, Spain, France, Israel, Italy, Luxembourg, Sweden, and Slovenia Mean age: 68 % Female: 57 Sample size: 40,797	Loneliness, UCLA-LS-3	NS	Face-to-Face Interview Surveys	NS	Cultural Practices	Age, Education, English Proficiency, Gender, Physical Limitations, Mental Health	Need more research to see if being from a collectivist country has the same effect on loneliness in younger people
(Garner et al. 2022) [53]	Frailty and mental well- through COVID-19 lockdowns	Country: UK, Spain % Female: 67.14 Sample size: 70	Loneliness, UCLA-LS-3	Social Isolation, Adult Social Care Outcomes Toolkit (ASCOT)	Face-to-Face Interview Surveys	Transport Access, Public Third-Places	NS	Age, Education, Income	No future research recommendations
(Settels, 2021) [78]	Neighbourhood conditions, the recession and the sizes of and turnover within social networks	Country: United States of America Mean age: 68.3 % Female: 51.31 Sample size: 1788	NS	Social Network, Single question 'Number of social ties'	Face-to-Face Interview Surveys	Neighbourhood Disadvantage	Social Security Recipients	Age, Chronic Condition, Education, Employment, Ethnicity, Gender, Income, Marital Status, Mental Health	Research older people in neighbourhoods that are experiencing changes
(Yang & Moorman, 2021) [84]	Neighbourhood trust, loneliness and number of friends	Country: United States of America Sample size: 5817	Loneliness, UCLA-LS-11	NS	Self-Complete Survey	Social Cohesion	Neighbourhood Belonging	Income, Marital Status, Physical Limitations	Intervention targeting neighbourhood trust and more longitudinal data
**Intervention**						Community Intervention	Societal Intervention		
**Pre-post Test**									
(Bartlett et al. 2013) [36]	Participation in three community programmes, levels of loneliness and social support	Country: Australia Mean age: 70.67 Sample size: 59	Loneliness, DJG-LS-11	NS	Self-Complete Survey	Three local interventions with local community services	NS	NS	Need more research on larger sample sizes and with more standardised controlled designs
(Bartsch & Rodgers, 2009) [37]	Senior Reach Gatekeeper Program outcomes and those of the established Spokane program	Country: United States of America % Female: 75 Sample size: 226	NS	Social Isolation, Tool Not Provided	Face-to-Face Interview Surveys	Community referral to a combination of available services, which may be mental health only, care management only, information and referral, or a combination	NS	NS	No future research recommendations
(Bartsch et al. 2013) [38]	Senior Reach Gatekeeper Program outcomes and those of the established Spokane program and the MKSO program	Country: United States of America % Female: 75 Sample size: 416	NS	Social Isolation, Tool Not Provided	Face-to-Face Interview Surveys	Community referral to a combination of available services, which may be mental health only, care management only, information and referral, or a combination	NS	NS	No future research recommendations
(Butler, 2006) [44]	The senior companion program	Country: United States of America Mean age: 78 % Female: 81.8 Sample size: 66	Loneliness, UCLA-LS-20	Social Network, LSNS-A-6	Face-to-Face Interview Surveys	Older volunteers complete in-home visits to less mobile older people	NS	NS	Need more research on how social workers might approach loneliness program evaluations
(Carandang et al. 2020) [46]	Community-based interventions to alleviate depressive symptoms	Country: Philippines Mean age: 68 % Female: 70.83 Sample size: 264	Loneliness, UCLA-LS-8	NS	Face-to-Face Interview Surveys	Peer counselling with 1-h weekly home visits, or to 3-h weekly social events held at a local senior centre, or both	NS	NS	Need more research on the long-term benefits of loneliness interventions
(Coll‐Planas et al. 2017) [47]	The intervention in mixed areas of diverse socioeconomic levels and to assess the effects on loneliness	Country: Spain Mean age: 77.24 % Female: 95 Sample size: 38	Loneliness, DJG-LS-11	NS	Face-to-Face Interview Surveys	A group-based program, promoting social participation among lonely older people	NS	NS	Clinical trials to provide evidence for a causal inference and for cost-effectiveness
(Gonyea & Burnes, 2013) [56]	Assistance for seniors, opportunities to build connections, feelings of loneliness or isolation, and aging-friendly communities	Country: United States of America Mean age: 81 % Female: 85 Sample size: 33	Loneliness, UCLA-LS-20	NS	Face-to-Face Interview Surveys	Community organisation providing services increasing access to existing community resources through outreach, education, advocacy, and providing transportation	NS	NS	Need more longitudinal studies that track participants of interventions
(Honigh-De Vlaming et al. 2013) [60]	Healthy Ageing in relation to loneliness	Country: Netherlands Mean age: 74 % Female: 56 Sample size: 858	Loneliness, DJG-LS-11	NS	Self-Complete Survey	NS	A mass media campaign, information meetings for interested local elderly people, psychosocial group courses for persons with mental health problems or chronic diseases, social activation by the community-based Neighbours Connected intervention, and training of intermediaries (homecare nurses, municipal advisors, and volunteers)	Age, Education, Gender, Income, Marital Status	Need more co-designed research with older people and the people delivering the interventions
(Merchant et al. 2021) [67]	Cognition amongst those at risk of isolation	Country: Singapore Sample size: 197	NS	Social Network, LSNS-6	Face-to-Face Interview Surveys	HAPPY- a dual-task exercise program adapted from cognicise, conducted in existing community sites by local volunteers	NS	Age	Need more commitment from sectors addressing older people’s health to implement healthy ageing initiatives
(Mulligan & Bennett, 1977) [69]	A resocialization program to reduce social isolation	Country: United States of America Mean age: 77 % Female: 91.3 Sample size: 23	NS	Social Isolation, Adulthood Isolation Index and Past Month Isolation Index	Face-to-Face Interview Surveys	Home visits from volunteers with an emphasis on friendly conversation	NS	NS	Program could conducts regular visits to older people to reduce their loneliness
(Mullins et al. 2020) [70]	A community-engaged, culturally informed technology program to address social isolation and loneliness	Country: United States of America Mean age: 74 Sample size: 262	Loneliness, UCLA-LS-20	NS	Self-Complete Survey	Internet provided to the older people's dwellings along with volunteers to run classes	NS	NS	Need longer evaluation periods for intervention studies like this one
(Passmore et al. 2007) [72]	The relationship between participating in community-based recreation activities and loneliness	Country: United States of America Mean age: 68.27 % Female: 56.67 Sample size: 30	Loneliness, UCLA-LS-10	NS	Face-to-Face Interview Surveys	Eight different Empowerment Groups built from existing community services	NS	NS	Interventions which control for the economic and social backgrounds of participants
(Reinhard et al. 2018) [73]	Social isolation, loneliness, and social engagement and transport use	Country: UK % Female: 54.7 Sample size: 18,164	Loneliness, UCLA-LS-3	Social Isolation, frequency of visits	Face-to-Face Interview Surveys	NS	Introduction of policy for an age-eligibility threshold for free bus travel	Age, Employment, Gender, Income, Marital Status, Physical Limitations, Religion, Children	No future research recommendations
(Roberts et al. 2020) [75]	A service designed to alleviate loneliness and isolation	Country: UK Mean age: 76.7 % Female: 81.7 Sample size: 120	Loneliness, DJG-LS-6	Social Isolation, LSNS-6	Face-to-Face Interview Surveys	Cadwyn Môn- Volunteer companionship, practical and psychological support	NS	NS	Need more trial style studies and an economic analysis of how cost-effective the intervention is
(Sandu et al. 2021) [77]	Using simple technology like telephone calls to reduce social isolation	Country: United States of America Sample size: 141	Loneliness, UCLA-LS-10	NS	Face-to-Face Interview Surveys	Community service agency paired student volunteers with their older adult clients in the community	NS	NS	Research effects of gender, age, socioeconomic status, ethnicity, and income on loneliness and social isolation
(Steinman et al. 2021) [79]	A program’s effect on social connectedness	Country: United States of America Mean age: 72.9 % Female: 79.1 Sample size: 320	Loneliness, UCLA-LS-3	NS	Face-to-Face Interview Surveys	Home-visits by trained front-line providers	NS	NS	Research how participants perceived age may affect their loneliness
(Toseland et al. 1979) [82]	Social isolation and obtaining needed social services	Country: United States of America % Female: 68.1 Sample size: 72	NS	Social Network, Tool Not Provided	Face-to-Face Interview Surveys	Peer counsellor home visits to assess the client's immediate social service needs. To help social relationships, networking techniques are used	NS	NS	Need more research on developing older people's self-help coping strategies and to foster social connections
**Randomised Control Trial**									
(Jones et al. 2019) [61]	A group exercise and socialisation/health education intervention and loneliness among those with hearing loss	Country: Canada Mean age: 74.5 % Female: 43 Sample size: 66	Loneliness, DJG-LS-11	NS	Face-to-Face Interview Surveys	Community organisation run program promoting socialisation	NS	NS	Need a larger sample size and more longitudinal data on this intervention
(Saito et al. 2012) [76]	A social isolation prevention program and loneliness	Country: Japan Mean age: 72 % Female: 65 Sample size: 63	Loneliness, Ando-Osada-Kodama (AOK) loneliness scale	Social Network, Single Question 'frequency of face-to-face contact with friends or neighbour’s’	Self-Complete Survey	NS	Group-based educational, cognitive, and social support program for improving community knowledge and networking with other participants. Included link workers to connect with services	NS	Need a larger sample size and to develop a variety of group-based programs targeting specific populations

*UCLA-LS* University of California Los Angeles Loneliness Scale, *DJG-LS* De Jong Gierveld Loneliness Scale, *LSNS* Lubben Social Network Scale, *NS* Not studied

### Publication date

The observational articles included in this review were published between 2001 and 2022, with a median year of publication of 2020, showing that the number of observational articles which are investigating the community and societal factors influencing loneliness and SI is growing each year, despite a dip in 2021. The interventional articles were published between 1977 and 2021, with two in the 1970’s [69, 82], and then none until 2006. There was a spike in interventional articles published in 2013, and again in 2020–21 [46, 67, 70, 75, 77, 79].

**Fig. 2:**
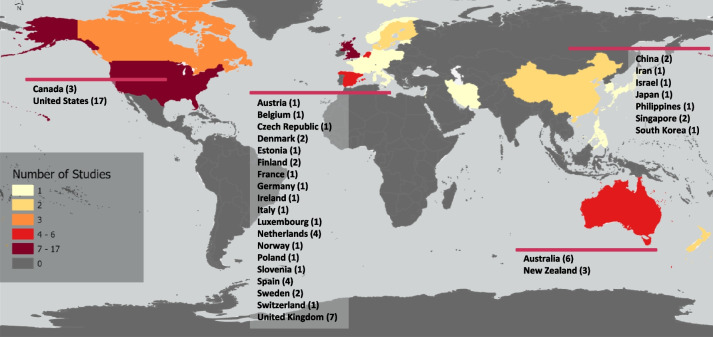
Global map showing the countries where the included articles data collection was conducted

The global spread of the included articles is depicted in Fig. 2, indicating a range of countries, with the most common being United States of America (USA) (*n* = 17) [34, 37, 38, 44, 45, 55, 56, 58, 66, 69, 70, 72, 77–79, 82, 84], the United Kingdom (UK) (*n* = 7) [39, 43, 50, 53, 73, 75, 83], and Australia (*n* = 6) [36, 40, 57, 59, 63, 64] (Table 2, Fig. 2). The majority of the interventions were conducted in the USA (*n* = 10) [37, 38, 56, 69, 70, 72, 77, 79, 82], with two in the UK [73, 75], and one each in Australia, Canada, Japan, Philippines, Spain, Singapore, and The Netherlands. Observational articles were more diversely spread, although still favoured predominantly English-speaking countries (*n* = 22) [34, 39–41, 43, 45, 49–51, 53–55, 57–59, 63, 64, 66, 78, 80, 83, 84].

### Data collection methods

The 52 included articles utilised quantitative surveys to collect data, which were either self-completed by participants (*n* = 17) [36, 39, 40, 45, 51, 55, 57, 60, 63, 66, 68, 70, 76, 80, 81, 83, 84], conducted as face-to-face interviews (*n* = 34) [34, 35, 37, 38, 41–44, 46–50, 52–54, 56, 58, 61, 62, 64, 65, 67, 69, 71–75, 77–79, 82, 85], or over the phone (*n* = 1) [59]. Interventional articles were more likely to detail the employment of face-to-face interview techniques compared to the other modalities (*n* = 15 face-to-face, *n* = 4 self-complete, *n* = 0 phone), as were observational articles (*n* = 19 face-to-face, *n* = 13 self-complete, *n *= 1 phone).

### Assessment of loneliness and social isolation

The main outcomes of interest for this scoping review were loneliness and SI. Of the 52 included articles, 65 per cent (*n* = 34) measured for loneliness only [35, 36, 39, 41–43, 45–49, 52, 54–58, 60–64, 66, 70–72, 74, 77, 79–81, 83–85], 13 per cent (*n* = 7) measured for SI only [37, 38, 65, 67, 69, 78, 82], and 21 per cent (*n* = 11) measured for both [34, 40, 44, 50, 51, 53, 59, 68, 73, 76, 77]. When measuring loneliness, the University of California, Los Angeles Loneliness Scale (UCLA-LS) and the De Jong Gierveld Loneliness Scale (DJG-LS) were the most commonly used, 35 per cent and 27 per cent (*n* = 18 and *n* = 14) of articles respectively. Among those using the UCLA-LS, there were seven different versions used, with the three-item scale the most commonly employed. Of the interventional articles measuring loneliness using the UCLA-LS, the longer 20-item scale was the most commonly used version. Similarly, for the articles using the DJG-LS to measure loneliness [36, 39, 43, 47, 49, 59–62, 68, 75, 80, 81, 85], the observational articles were more likely to utilise the shorter six-item version (*n* = 6) [43, 49, 59, 80, 81, 85], while the interventional articles were more likely to utilise the longer 11-item version (*n* = 4) [36, 47, 60, 61]. Single-item questions to measure loneliness were the next most commonly used tool (*n* = 10), but were exclusively used in cross-sectional design, although the wording varied: seven of 10 articles including a variant of the word lonely, for example ‘lonely’ or ‘loneliness’ [34, 35, 48, 50, 63, 64, 83], with the remaining three using ‘isolated’ or ‘disconnected’ to capture participants’ subjective isolation [40, 41, 45].

Of the 18 articles measuring objective isolation, five used the Lubben Social Network Scale (LSNS). Four of the five articles that detailed the use of the LSNS, including three interventional studies, used the shorter six-item version [44, 65, 67, 75], while one observational article used the longer 12-item scale version [51]. There was heterogeneity regarding how the articles named the variable in question with three of the five articles using the LSNS stated that they were measuring ‘Social Network’ using the scale [44, 51, 67], while one called it ‘Social Health’ [65], and another named the variable ‘Social Isolation’ [75]. Three articles measuring objective isolation opted for the use of a single-item question, asking participants directly how many friends and family they have, or the frequency of their social visits. The questions used were ‘state the number of friends and family members you have’ [59], ‘how many social ties do you have?’ [78], and ‘what is the frequency of face-to-face contact with friends or neighbours?’ [76]. A further three articles did not state their measurement tool, two of which measured ‘Social Isolation’ and one measured ‘Social Network’ [37, 38, 82]. The remaining seven articles used a range of other techniques which can be seen in Table 2.

### Summary of community and societal factors investigated by included observational studies

Eighteen community and societal factors were considered as influential factors on loneliness, SI, or both (Table 3). Variables were classified as a community- or societal- level factors depending on the context in which they were operationalised in the primary study, the level of government that would be required to enact change over the factor, and the scope of the impact of the factor. The *community* level factors were (see definitions in Table 3): Neighbourhood disadvantage, open green spaces, accessible services, neighbourhood density, neighbourhood satisfaction, rurality, social cohesion, walkability, transport access, neighbourhood safety, and public third-places. These were determined as community factors due to their influence on the local environment and the fact that they affect a localised group of people rather than the broader society. The *societal* factors were (see definitions in Table 3): Housing diversity, political participation, perceptions of ageism, social security recipients, migration, neighbourhood belonging, and cultural practices. These were determined to be societal-level factors due to their dependence on social and economic policy, as well as the shared ideas and beliefs of the broader area (country or otherwise) in which the community is placed.

**Table 3:** The community and societal factors, their definitions and their associations with loneliness and social isolation

Factor (Classification)	Definition	Association with loneliness (Source)	Association with social isolation (Source)
Neighbourhood Disadvantage (Community)	The socioeconomic status of the area	Positive association (Lam and Wang 2022) [64]	Positive association (Settels 2021) [78]
Open Green Space (Community)	The amount of non-vegetated green space in the community	Negative association (Lam and Wang 2022; Park et al. 2021) [64, 71]	Not studied
Accessible Services (Community)	Accessibility to community buildings and infrastructure	Negative association (Gibney et al. 2019; Hodgkin et al. 2018; Stephens and Phillips 2022) [63, 64]	Negative association (Hodgkin et al. 2018; Stephens and Phillips 2022) [59, 80]
Neighbourhood Density (Community)	Population density of neighbourhood	Negative association (Lam 2022; Lam and Wang 2022) [63, 64]	Negative association (Lane et al. 2020) [65]
Neighbourhood Satisfaction (Community)	Resident-rated satisfaction with neighbourhood	Negative association (Glass 2020; Hodgkin et al. 2018; Lam 2022) [55, 59, 63]	Negative association (Hodgkin et al. 2018) [59]
Rurality (Community)	Geographic classification of area	Positive association (Beer et al. 2016; Beere et al. 2019; Henning‐Smith et al. 2019) [40, 41, 58]	Positive association (Beer et al. 2016) [40]
Social Cohesion (Community)	How supportive the neighbourhood is, as rated by participants	Negative association (Bai et al. 2021; Stephens and Phillips 2022; Yang and Moorman 2021) [35, 80, 84]	Negative association (Stephens and Phillips 2022) [80]
Walkability (Community)	Ease of walking around neighbourhood and to third places	Negative association (Domenech-Abella et al. 2020; Gibney et al. 2019; Park et al. 2021) [52, 54, 71]	Negative association (Lane et al. 2020) [65]
Transport Access (Community)	Public transport availability	Negative association (De Koning et al. 2017; Gibney et al. 2019; Park et al. 2021; Rezaeipandari et al. 2020; Woolham et al. 2013) [50, 54, 71, 74, 83]	Negative association (Garner et al. 2022) [53]
Public Third Places (Community)	Provision of places in the community in which social activities can occur, outside of people’s usual home and work spaces	Negative association (Cao et al. 2020; Lee 2022; Park et al. 2021; Rezaeipandari et al. 2020) [45, 66, 71, 74], No effect (Moorer and Suurmeijer 2001) [68]	Negative association (Domenech-Abella et al. 2020; Lane et al. 2020) [52, 65], No effect (Moorer and Suurmeijer 2001) [68]
Neighbourhood Safety (Community)	Perceived and actual crime in neighbourhood	Negative association (Dahlberg et al. 2022; Hodgkin et al. 2018; Lam 2022; Park et al. 2021; Rezaeipandari et al. 2020; Stephens and Phillips 2022; Woolham et al. 2013; Zhang and Lu 2022) [48, 59, 63, 71, 74, 80, 83, 85], No effect (Moorer and Suurmeijer 2001; Timmermans et al. 2021) [68, 81]	Negative association (Garner et al. 2022; Hodgkin et al. 2018; Stephens and Phillips 2022) [53, 59, 80], No effect (Moorer and Suurmeijer 2001) [68]
Housing Diversity (Societal)	Diversity of types of housing because of zoning	Negative association (Lam and Wang 2022) [64]	Not studied
Political Participation (Societal)	Participation in civic activities in countries where this is not mandatory	Negative association (Dahlberg et al. 2022; Henning‐Smith et al. 2019) [48, 58]	Not studied
Perceptions Of Ageism (Societal)	Perceived ageism by participants	Negative association (Gibney et al. 2019; Hodgkin et al. 2018; Park et al. 2021) [54, 59, 71]	Negative association (Hodgkin et al. 2018) [59]
Social Security Recipients (Societal)	Proportion of neighbourhood receiving social security benefits/pensions	Positive association (Timmermans et al. 2021) [81]	Positive association (Settels 2021) [78]
Migration (Societal)	Migrant status of participant	Positive association (Ajrouch 2008; De Jong Gierveld et al. 2015; Henning‐Smith et al. 2019; Klok et al. 2017) [34, 49, 58, 62]	Positive association (Ajrouch 2008) [34]
Neighbourhood Belonging (Societal)	Self-rated perception of belonging to the neighbourhood	Negative association (Beech and Murray 2013; Glass 2020; Stephens and Phillips 2022; Zhang and Lu 2022) [39, 55, 80, 85]	Negative association (Stephens and Phillips 2022; Yang and Moorman 2021) [80, 84]
Cultural Practices (Societal)	The material factors of culture such as taking part in cultural activities	Negative association (Beller and Wagner 2020; Burholt et al. 2018; De Jong Gierveld et al. 2015; Diaz et al. 2019; Haslam et al. 2022; Klok et al. 2017) [42, 43, 49, 51, 57, 62], No effect (Ajrouch 2008) [34]	Negative association (Diaz et al. 2019) [51]

**Fig. 3:**
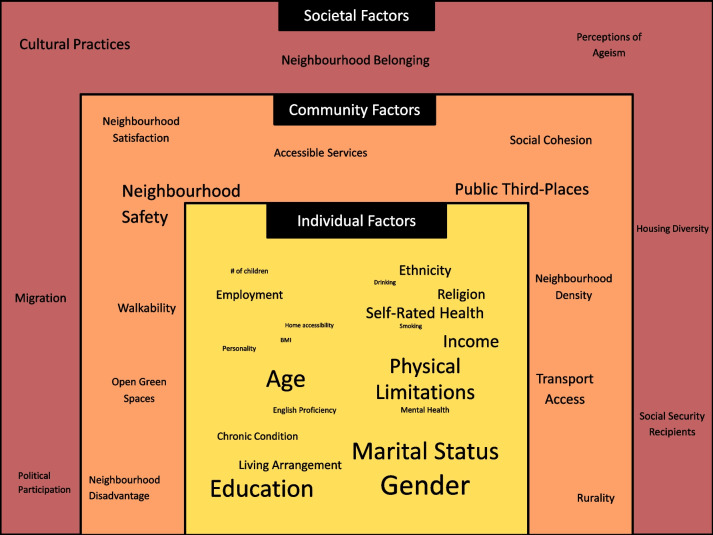
The individual, community and societal factors investigated in the observational articles Notes: Size of the font denotes the number of articles using the factor in their analysis

Of the 33 observational studies, 36 per cent (*n* = 12) of articles investigated both community and societal factors [49, 54, 55, 58, 63, 64, 71, 78, 80, 81, 84, 85], while 39 per cent (*n* = 13) investigated only community level factors [35, 40, 41, 45, 50, 52, 53, 59, 65, 66, 68, 74, 83], and 24 per cent (*n* = 8) investigated only societal level factors [34, 39, 42, 43, 48, 51, 57, 62]. The most investigated community factors (Fig. 3) were neighbourhood safety (*n* = 10) [48, 59, 63, 68, 71, 74, 80, 81, 83, 85], public third-places (*n* = 9) [45, 52, 53, 65, 66, 68, 71, 74, 81], and transport access (*n* = 6) [50, 53, 54, 71, 74, 83], while the most investigated societal factors were cultural practices (*n* = 7) [34, 42, 43, 49, 51, 57, 62], neighbourhood belonging (*n* = 5) [39, 55, 80, 84, 85], and migration (*n* = 4) [34, 58, 62, 63]. There was no difference in the factors investigated according to whether the outcome being measured was loneliness, SI, or both.

### The association between community factors, societal factors, loneliness and social isolation

Positive associations were found for neighbourhood disadvantage and loneliness and SI. Similarly for rurality, migration, and social security recipients. Negative associations were found between loneliness and open green spaces, accessible services, neighbourhood density, neighbourhood satisfaction, social cohesion, walkability, transport access, housing diversity, political participation, perceptions of ageism, migration and neighbourhood belonging, there were mixed findings for the association between loneliness and cultural practices, neighbourhood safety, and public third-places with each factor found either a negative association or no influence on loneliness. SI was found to have a negative association with accessible services, neighbourhood density, neighbourhood satisfaction, social cohesion, walkability, transport access, perceptions of ageism, neighbourhood belonging, and cultural practices. There were mixed results for the association between SI and neighbourhood safety and public third-places, with both having either a negative association or no influence on SI.

### Community and societal factor associations

#### Intervention approaches to community and societal influences of loneliness and social isolation

There were 19 articles which detailed an intervention to reduce loneliness or social isolation. Of those 84 per cent (*n* = 16) were community level interventions [36–38, 44, 46, 47, 56, 61, 67, 69, 70, 72, 75, 77, 79, 82], while 16 per cent (*n* = 3) were societal level interventions [60, 73, 76]. The community level interventions typically involved the use of one of two approaches. The first is the involvement of community volunteers who act as manufactured connections for the older participants as a part of the program, of which there were nine articles detailing this approach, all using pre-post test methods [44, 46, 67, 69, 70, 75, 77, 79, 82]. These interventions were considered to be community-level rather than individual as there was a potential, due to their involvement of community volunteers, for the social engagement to continue outside the confines of the study environment. The second is the education of the older participants about what services and activities already exist in their community and potentially connecting them with these services. There were seven articles detailing this approach, six pre-post test articles, and one RCT [36–38, 47, 56, 61, 72]. Of the societal level interventions, one incorporated a mass media campaign to reduce ageism in the community as a part of the intervention measuring the changes in loneliness [60]. Another intervention changed the perceptions of older people by specifically training volunteer members of the community in how to reduce ageism, and measured the changes in loneliness and social networks [76]. The other measured changes in loneliness and SI as a result of the introduction of free public transport in the UK for older people [73].

#### Individual and interpersonal factors as covariates

There were 21 covariates identified from the 52 included articles that are known micro-level individual and interpersonal factors of loneliness and SI as can be seen in Table 2. The most commonly adjusted for factors were gender (*n* = 24), age (*n* = 23), marital status (*n* = 21), and education (*n* = 20). The least used micro-level factors were home accessibility, smoking status and drinking status (*n* = 1) [35, 45]. All the observational articles stated the covariates used for analysis, with 81 per cent (*n* = 27) using more than two co-variates in their analysis [34, 35, 42, 43, 45, 48, 49, 51–54, 57–59, 62–66, 71, 74, 78, 80, 81, 83–85]. Only three interventional articles stated which covariates were used [60, 67, 73].

#### Quality of the included studies

In the risk of bias assessment, 17 articles were identified as having high quality [43, 45, 48–50, 52, 54, 57, 59, 62, 64–66, 74, 80, 81, 85], while four were identified as having very low quality [36, 40, 44, 77], as can be seen in Table 4. Any conclusions drawn using the articles of very low quality should be done so with caution. It is not recommended that further aggregating of the risk of bias scores be undertaken, and an assessment of those with high and very low quality is shown to make interpretation clearer.

**Table 4:** Critical appraisal of all included studies using design specific JBI critical appraisal tools

Study type	Source		Appraisal															
Observation studies																		
Cross sectional			Checklist for analytical cross-sectional studies															
			Inclusion Criteria Defined		Study subjects and the setting described		Exposure measured in a valid and reliable way		Objective, standard criteria used for measurement of the condition		Confounding factors identified			Strategies to deal with confounding factors stated	Outcomes measured in a valid and reliable way			Appropriate statistical analysis used
	(Ajrouch 2008)	[34]	Yes		Yes		Yes		Yes		Yes			Yes	Yes			Unsure
	(Bai et al. 2021)	[35]	Yes		Yes		Unsure		Yes		Yes			Yes	Unsure			Yes
	(Beech and Murray 2013)	[39]	Yes		Yes		Yes		Yes		No			No	Yes			No
	(Beer et al. 2016)^b^	[40]	No		No		Yes		Yes		No			No	Yes			No
	(Beere et al. 2019)	[41]	Yes		No		Yes		Yes		No			No	Yes			No
	(Burholt et al. 2018)^a^	[43]	Yes		Yes		Yes		Yes		Yes			Yes	Yes			Yes
	(Cao et al. 2020)^a^	[45]	Yes		Yes		Yes		Yes		Yes			Yes	Yes			Yes
	(Dahlberg et al. 2022)^a^	[48]	Yes		Yes		Yes		Yes		Yes			Yes	Yes			Yes
	(De Jong Gierveld et al. 2015)^a^	[49]	Yes		Yes		Yes		Yes		Yes			Yes	Yes			Yes
	(De Koning et al. 2017)^a^	[50]	Yes		Yes		Yes		Yes		Yes			Yes	Yes			Yes
	(Diaz et al. 2019)	[51]	No		Yes		No		Yes		Yes			Yes	No			Yes
	(Domenech-Abella et al. 2020)^a^	[52]	Yes		Yes		Yes		Yes		Yes			Yes	Yes			Yes
	(Gibney et al. 2019)^a^	[54]	Yes		Yes		Yes		Yes		Yes			Yes	Yes			Yes
	(Glass 2020)	[55]	Yes		Yes		Yes		Yes		No			No	Yes			Yes
	(Haslam et al. 2022)^a^	[57]	Yes		Yes		Yes		Yes		Yes			Yes	Yes			Yes
	(Henning‐Smith et al. 2019)	[58]	Yes		Yes		Yes		Yes		Yes			No	Yes			Unsure
	(Hodgkin et al. 2018)^a^	[59]	Yes		Yes		Yes		Yes		Yes			Yes	Yes			Yes
	(Klok et al. 2017)^a^	[62]	Yes		Yes		Yes		Yes		Yes			Yes	Yes			Yes
	(Lam and Wang 2022)^a^	[64]	Yes		Yes		Yes		Yes		Yes			Yes	Yes			Yes
	(Lam 2022)	[63]	Yes		No		Yes		Yes		Yes			Yes	Yes			Unsure
	(Lane et al. 2020)^a^	[65]	Yes		Yes		Yes		Yes		Yes			Yes	Yes			Yes
	(Lee 2022)^a^	[66]	Yes		Yes		Yes		Yes		Yes			Yes	Yes			Yes
	(Moorer and Suurmeijer 2001)	[68]	Yes		Yes		Yes		Unsure		Yes			Yes	Yes			Yes
	(Park et al. 2021)	[71]	Yes		Yes		Unsure		Yes		Yes			Yes	Unsure			Yes
	(Rezaeipandari et al. 2020)^a^	[74]	Yes		Yes		Yes		Yes		Yes			Yes	Yes			Yes
	(Stephens and Phillips 2022)^a^	[80]	Yes		Yes		Yes		Yes		Yes			Yes	Yes			Yes
	(Timmermans et al. 2021)^a^	[81]	Yes		Yes		Yes		Yes		Yes			Yes	Yes			Yes
	(Woolham et al. 2013)	[83]	Yes		Yes		Yes		Yes		No			No	Yes			No
	(Zhang and Lu 2022)^a^	[85]	Yes		Yes		Yes		Yes		Yes			Yes	Yes			Yes
Cohort			Checklist for cohort studies															
			Groups similar and recruited from the same population		Exposures measured similarly to assign groups	Exposure measured in a valid and reliable way	Confounding factors identified		Strategies to deal with confounding factors stated		Participants free of the outcome at the start of the study		Outcomes measured in a valid and reliable way	Adequate follow up time for outcome to occur	Follow up complete	Strategies to address incomplete follow up used		Appropriate statistical analysis used
	(Beller and Wagner 2020)	[42]	N/A		N/A	Yes	Yes		Yes		No		Yes	Yes	Yes	Yes		Yes
	(Garner et al. 2022)	[53]	N/A		N/A	Yes	Yes		Yes		No		Yes	Yes	No	No		Yes
	(Settels 2021)	[78]	N/A		N/A	Yes	Yes		Yes		No		Yes	Yes	Yes	Yes		Yes
	(Yang and Moorman 2021)	[84]	N/A		N/A	Yes	Yes		Yes		No		Yes	Yes	Yes	N/A		Yes
**Intervention Studies**																		
Pre-post test																		
	Source		Clear what is the cause’ and what is the ‘effect’		Participants included in any comparisons similar	Participants included in any comparisons receiving similar treatment/care	Inclusion of control group		Multiple measurements of the outcome both pre and post the intervention/exposure				Follow up complete		Outcomes of participants included in any comparisons measured in the same way		Outcomes measured in a reliable way	Appropriate statistical analysis used
	(Bartlett et al. 2013)^b^	[36]	Yes		Yes	Unsure	No		No				Yes		Yes		No	No
	(Bartsch and Rodgers 2009)	[37]	Yes		No	Yes	No		No				Yes		Yes		Unsure	Yes
	(Bartsch et al. 2013)	[38]	Yes		No	Yes	No		No				Yes		Yes		Unsure	Yes
	(Butler 2006)^b^	[44]	No		Yes	No	No		No				No		Yes		Unsure	No
	(Carandang et al. 2020)	[46]	Yes		Yes	Yes	Yes		No				Yes		Yes		Yes	Yes
	(Coll‐Planas et al. 2017)	[47]	Yes		Yes	Yes	No		No				No		Yes		Yes	Yes
	(Gonyea and Burnes 2013)	[56]	No		Yes	Yes	No		No				Yes		Yes		Unsure	Yes
	(Honigh-De Vlaming et al. 2013)	[60]	Yes		Yes	Yes	Yes		No				Yes		Yes		Yes	Yes
	(Merchant et al. 2021)	[67]	Yes		Yes	Yes	No		No				Yes		Yes		Unsure	Yes
	(Mulligan and Bennett 1977)	[69]	Yes		Yes	Yes	Yes		No				Yes		Yes		No	Yes
	(Mullins et al. 2020)	[70]	Yes		Yes	Yes	No		No				Unsure		Yes		Yes	Yes
	(Passmore et al. 2007)	[72]	Yes		Yes	Yes	No		No				Yes		Yes		Yes	No
	(Reinhard et al. 2018)	[73]	Yes		No	Yes	Yes		No				Yes		Yes		Yes	Yes
	(Roberts et al. 2020)	[75]	Yes		Yes	Yes	No		No				Yes		Yes		Yes	Yes
	(Sandu et al. 2021) ^b^	[77]	Yes		Yes	No	No		No				No		Yes		No	Yes
	(Steinman et al. 2021)	[79]	No		Yes	Yes	No		No				Yes		Yes		Unsure	Yes
	(Toseland et al. 1979)	[82]	Yes		Yes	Yes	No		No				Yes		Yes		Yes	Yes
Randomised control trials			Checklist for randomised controlled trials															
			True randomisation used for assignment of participants	Allocation to treatment groups concealed	Treatment groups similar at the baseline	Were participants blind to treatment assignment	Those delivering treatment blind to treatment assignment	Were outcomes assessors blind to treatment assignment?		Treatment groups treated identically other than the intervention		Follow up complete	Participants analysed in the groups to which they were randomised	Outcomes measured in the same way for treatment groups	Outcomes measured in a reliable way	Appropriate statistical analysis used		Appropriate trial design used
	(Jones et al. 2019)	[61]	Yes	No	Yes	Yes	No	No		Yes		Yes	Yes	Yes	Yes	Yes		Yes
	(Saito et al. 2012)	[76]	Yes	No	Yes	No	No	No		No		Yes	Yes	Yes	Yes	Yes		Yes

^a^High quality articles > 90 per cent,

^b^low quality articles < 50 per cent. Questions edited for brevity

#### Critical appraisal

Most (*n* = 46) of the included articles included recommendations for future research based on their results [34–36, 39, 41–52, 54–67, 69–72, 74–85]. The need for more longitudinal data was raised explicitly in eight articles, six of which were observational, and two intervention articles recommended that future interventions should have more comprehensive longitudinal follow-up periods [35, 48, 52, 54, 56, 61, 71, 84]. Another common recommendation was for there to be more research investigating the effects of loneliness and SI on migrant populations, all of which were cross-sectional in design [34, 43, 57, 62, 63]. One article recommended that future qualitative research on underserved populations would be beneficial to understanding the factors affecting loneliness [83]. A recent comprehensive systematic review by C Noone and K Yang [86]details the current state of qualitative research addressing community level factors influencing loneliness in older people and is a good resource.

## Discussion

This scoping review examined how the community- and societal- level factors of loneliness and SI are being researched in older populations. We explored the methodology used in the existing research, and determined which factors were being investigated. We posit reasons for the most and least explored variables and provide recommendations for future research. Our results show that there is a growing body of research in the field of loneliness and SI, particularly since 2020, addressing community and societal factors of loneliness and SI, whether researchers explicitly mentioned the social-ecological framework, or it was implied from the data they collected. There does appear to be some confusion, however, surrounding the concept of SI with differing definitions and terms used to describe similar experiences. Research focused on community factors more commonly compared to societal factors. Within these categories the most researched factors were neighbourhood safety and cultural practices respectively, and the least researched factors were neighbourhood disadvantage, open green spaces, housing diversity, and political participation. However, current knowledge is largely based on correlational studies from English-speaking countries. There is a paucity of longitudinal studies and well-designed interventional studies, with loneliness and SI as the main outcome, as a result there is insufficient empirical research to address these health issues through more upstream systemic drivers.

An important consideration in current loneliness and SI research is the potential impact of the global COVID-19 pandemic which began in late 2019, reaching its global peak in 2020. Of the 25 studies that were published during the pandemic, only four detailed data collection that was completed during or after 2019 [53, 77, 79, 85]. Of these four articles, only one aimed to investigate the impacts of the socialisation policies such as lockdowns on loneliness and SI experienced by older people, finding little to no effect from lockdown protocols on loneliness and SI [53]. One article determined that their results were not affected due to the COVID-19 pandemic due to data being collected post-lockdowns [85]. The other two articles took the impact of the global pandemic into consideration while completing their analyses, but as this was not the aim of their studies, the authors opted to only comment briefly on the pandemic [77, 79]. Both followed a pre-post test design and found no negative impact from COVID-19 on the effectiveness of their intervention, with one finding no effect as a result of the pandemic [79], and one finding a small positive effect [77].

The societal and community factors were more comprehensively addressed in loneliness research compared with SI; most community and societal factors were investigated by at least one article in relation to their influence on loneliness. Community factors were more likely to be investigated in relation to SI than the societal factors, with only three societal factors thus far having been investigated, namely cultural practices, migration and receipt of social security. These three are societal factors which have also been investigated for other social phenomena topics such as violence and health service utilisation [21, 87]. Community factors including open green spaces and social cohesion were not investigated in relation to SI, which is surprising given the importance of open green space to social integration, sense of community and facilitation of acculturation of ethnic minorities or marginalised groups [88–90]. More research is needed to determine whether the influence of open green spaces does extend to SI. Similarly, it is surprising that social cohesion has not been investigated in relation to SI among older people. A significant association between social cohesion and SI has been found in a sample of younger people, although the causality of this relationship cannot be expanded further, heralding a need for further research to assess the association, as well as further research in an older sample [91].

Across all the factors investigated in the observational studies, the community factors, in particular neighbourhood safety and public third-places were the most commonly investigated. Neighbourhood safety, including both perceived and actual crime rates, appears to be an important determinant of loneliness and SI with articles suggesting that the fear of crime may increase the rates of loneliness, particularly in men [92]. One potential mechanism for the connection between neighbourhood safety and loneliness is through the decreased time spent leisure walking around the neighbourhood when perceived neighbourhood safety is low, which in turn decreases the number of opportunities for finding social connections, thereby increasing loneliness [93]. Similarly, the literature is in agreeance that increased neighbourhood provisions of public third-places, such as community centres and libraries, where older people can safely engage with each other will likely decrease both loneliness and SI [94]. Increased provisions of public third-places may also be a factor that influences whether older people opt for home-based care rather than entering retirement villages, and nursing homes as conduits for social activity, and social group activities [95]. This may bias our results, as we did not include articles detailing older people residing within care services in this review, thereby increasing the likelihood of public third spaces being important factors for influence over loneliness and SI [87].

Within the included observational studies, each community- and societal- level factor was investigated by more than one article except for political participation and housing diversity which were investigated by one article each, and are both societal level factors. Political participation, in particular voting behaviour, is difficult to explore on the global scale due to differing legislation across the world, with some countries mandating voting participation, while in others, like the USA, participation is not mandated and is more likely to be determined by societal norms and the desire for social conformity [96, 97]. Previous articles have described potential connections between loneliness and voting behaviour, with a need for more research in this area [98]. In a German and Dutch sample, it was found that there is reverse causality between civic duty and voting behaviour, with people who are lonely having a lower sense of civic duty as a result of their detachment from society, and were therefore less likely to partake in political voting [98]. Housing diversity is a result of zoning legislation put in place by governments and can affect the social health of communities [99]. For example, urbanisation of communities can be detrimental to the social health of its constituents by encouraging gentrification, where it is usual for a mass exodus from the neighbourhood to occur, thereby causing the loss of community ties, especially in those who have lived in the community for an extended period of time [100]. Older people generally rely on neighbourhood ties for a range of things which help them to remain independent, meaning that when they lose these important ties they also lose their social ties [101].

It was common for the included articles to use a single question to determine loneliness or SI, perhaps to reduce participant burden. The validity of a single-item question compared to a validated scale depends on the question being used. In terms of loneliness, previous work has determined that there is little difference when comparing the question ‘how often are you lonely?’ to the validated UCLA-three item scale [102]. ‘How often are you lonely?’ is also the preferred single-item question recommended by the Campaign to End Loneliness [103]. Measuring SI with a single-item question was less common, with only three of the included articles using this measure [59, 76, 78]. The questions used included ‘number of friends and family members?’, ‘how many social ties do you have?’, and ‘what is the frequency of face-to-face contact with friends or neighbours?’. It remains unclear as to whether a single-item question is sufficient to measure SI, with researchers unable to ascertain both the network size and frequency of contact in a singular question [7].

We also report in our results method of data collection and found this is primarily face-to-face interviews or self-complete questionnaires. The internal reliability of these methods has been questioned before, as loneliness and SI are still socially stigmatised topics [104]. In general, when participants rate the subject content of a question as sensitive they are more likely to under-report the outcome during a face-to-face interview compared to when asked with a self-complete questionnaire [105]. While face-to-face interviews may introduce some measurement bias to the findings in relation to the strength and significance of the association, it is unlikely to impact the direction of the association which we reported here.

The global spread of studies investigating the community and societal factors of loneliness and SI for older people is limited, with no research in the African and South American continents, consistent with conclusions from other research not just in older people, but across the life course [10]. The included articles detailed studies that were conducted primarily in countries which are member states of the Organisation for Economic Co-operation and Development (OECD) [106]. Twenty-six of the 30 included countries are member states of the OECD. This bias may be due the fact that the OCED establish international standards and advise member states on public policy and highlights the inequalities in social indicators, including social connection which may encourage research on the social welfare of the populations [106].

A limitation found in the current available evidence that impacts the inter-reliability of articles is the heterogeneity of the terms used in articles, even if definitions do not differ and the measurement tool itself is the same. We found that for SI, known to be the objective isolation of participants, there were three different terms used across the 18 relevant articles which were, namely ‘social isolation’, ‘social network’, and ‘social health’. The issue of inconsistency in the measurement of SI has been identified in previous reviews [7, 13]. Of the articles included in our review using the Lubben Social Network Scale (LSNS) to measure SI, three articles labelled the outcome ‘social network’, one labelled it ‘social isolation,’ and another labelled it social health. The authors of the original LSNS article state that it is to be used as a tool to screen for SI in older people [107]. The variation can lead to the duplication of research, which is slowing potential progress in this important topic and so a clear definition of SI is needed in the literature [7].

This review found that community level factors and interventions were addressed more commonly than societal level factors and interventions. These were both characterised by more opportunistic approaches, for example, as a result of government policy changes that were picked up by researchers for evaluation, or large amounts of funding. With community-level interventions, half relied entirely on community volunteers for the execution of their interventions, rather than building a systematic intervention which involves volunteers.

Significant grass-roots work is being undertaken, providing community-based solutions to the problems of loneliness and SI. Yet with evaluation taking place through policy makers and community organisations, the findings may not be publicly available and therefore are less likely to be translated to other populations [108]. Similarly, there are a number of not-for-profit organisations particularly in OECD countries, aiming to reduce the burden of loneliness and SI. Whether their impact will be demonstrated in the literature remains to be seen.

### Strengths and limitations of the scoping review

A strength of this review is the large number of included articles containing both intervention and observational methodologies. It is, to the best of the authors’ knowledge, the first scoping review to ascertain how the research archive has captured the influence of community and societal factors on both loneliness and SI in older people. In doing this, the comparison between objective SI and subjective loneliness is another strength, as the two are strongly related and therefore should be investigated in tandem [109]. The risk factors and health implications for loneliness and SI found in the literature are very similar, and so, by researching loneliness and SI in tandem, it allows for a more comprehensive overview of a population [8].

A limitation of this review is the exclusion of qualitative studies, from which further insights may be drawn. This exclusion was made to narrow the scope of this review to be manageable within the time and resources allocated, but future research should be undertaken to investigate the qualitative perspectives of the influence of community and societal factors on loneliness and SI. Understanding the lived experience of older people experiencing loneliness and SI is important to inform interventions and to understand the underlying mechanisms through which we can effect change [110, 111].

This review has found a growing research archive investigating the community and societal influences of loneliness and SI in older people, however, there are still gaps in the knowledge. There is a clear lack of high-quality longitudinal data, which is needed to infer causality between the influential community and societal factors and loneliness and SI in older people [20, 52]. There is also a need for more research about how these factors may influence SI as this concept was less likely to be explored. Finally, there is a need for qualitative perspectives to be explored to gain a deeper understanding on the way that older people may experience the effect of community and societal factors on loneliness and SI [83]. Future studies should more explicitly explore the community and societal factors of loneliness and SI in older people to further develop the evidence base [49].

A lack of research observing certain community- and societal-level factors means that few conclusions can be drawn about their influence over loneliness and SI. It is hoped that by bringing attention to the social-ecological approach to loneliness and SI research in older people there will be an increased awareness by researchers to expand and evaluate this topic. An improved understanding of the relationships and mechanisms through which community- and societal- level factors affect loneliness and SI can also be tested in future interventions. A social-ecological approach to loneliness and SI appears to be feasible, and further research, including more longitudinal and qualitative studies will serve to guide effective solutions to reduce loneliness and SI in our older populations.

## Data Availability

All data generated or analysed during this study are included in this published article.

## Abbreviations

SI: Social Isolation
PCC: Population, concept and context
USA: United States of America
UK: United Kingdom
UCLA-LS: University of California, Los Angeles Loneliness Scale
DJG-LS: De Jong Gierveld Loneliness Scale
LSNS: Lubben Social Network Scale
OCED: Organisation for Economic Co-operation and Development
